# Diastolic Ventricular Interaction in Heart Failure With Preserved Ejection Fraction

**DOI:** 10.1161/JAHA.118.010114

**Published:** 2019-03-29

**Authors:** Sathish K. Parasuraman, Brodie L. Loudon, Crystal Lowery, Donnie Cameron, Satnam Singh, Konstantin Schwarz, Nicholas D. Gollop, Amelia Rudd, Fergus McKiddie, Jim J. Phillips, Sanjay K. Prasad, Andrew M. Wilson, Srijita Sen‐Chowdhry, Allan Clark, Vassilios S. Vassiliou, Dana K. Dawson, Michael P. Frenneaux

**Affiliations:** ^1^ Norwich Medical School University of East Anglia Norwich United Kingdom; ^2^ Royal Infirmary of Edinburgh United Kingdom; ^3^ Royal Stoke University Hospital Stoke‐on‐Trent United Kingdom; ^4^ Department of Cardiology School of Medicine & Dentistry University of Aberdeen United Kingdom; ^5^ Nuclear Medicine Aberdeen Royal Infirmary NHS Grampian Aberdeen United Kingdom; ^6^ Royal Brompton Hospital and Imperial College London London United Kingdom; ^7^ Institute of Cardiovascular Science University College London London United Kingdom

**Keywords:** diastolic ventricular interaction, exercise pulmonary hypertension, heart failure, Heart Failure, Physiology, Clinical Studies, Contractile function, Hemodynamics

## Abstract

**Background:**

Exercise‐induced pulmonary hypertension is common in heart failure with preserved ejection fraction (HFpEF). We hypothesized that this could result in pericardial constraint and diastolic ventricular interaction in some patients during exercise.

**Methods and Results:**

Contrast stress echocardiography was performed in 30 HFpEF patients, 17 hypertensive controls, and 17 normotensive controls (healthy). Cardiac volumes, and normalized radius of curvature (NRC) of the interventricular septum at end‐diastole and end‐systole, were measured at rest and peak‐exercise, and compared between the groups. The septum was circular at rest in all 3 groups at end‐diastole. At peak‐exercise, end‐systolic NRC increased to 1.47±0.05 (*P*<0.001) in HFpEF patients, confirming development of pulmonary hypertension. End‐diastolic NRC also increased to 1.54±0.07 (*P*<0.001) in HFpEF patients, indicating septal flattening, and this correlated significantly with end‐systolic NRC (ρ=0.51, *P*=0.007). In hypertensive controls and healthy controls, peak‐exercise end‐systolic NRC increased, but this was significantly less than observed in HFpEF patients (HFpEF,* P*=0.02 versus hypertensive controls; *P*<0.001 versus healthy). There were also small, non‐significant increases in end‐diastolic NRC in both groups (hypertensive controls, +0.17±0.05, *P*=0.38; healthy, +0.06±0.03, *P*=0.93). In HFpEF patients, peak‐exercise end‐diastolic NRC also negatively correlated (*r*=−0.40, *P*<0.05) with the change in left ventricular end‐diastolic volume with exercise (ie, the Frank‐Starling mechanism), and a trend was noted towards a negative correlation with change in stroke volume (*r*=−0.36, *P*=0.08).

**Conclusions:**

Exercise pulmonary hypertension causes substantial diastolic ventricular interaction on exercise in some patients with HFpEF, and this restriction to left ventricular filling by the right ventricle exacerbates the pre‐existing impaired Frank‐Starling response in these patients.

**Clinical Perspective**

**What Is New?**

- This study demonstrates that diastolic ventricular interaction develops with exercise in a non‐obese sub‐phenotype of patients with heart failure with preserved ejection fraction.
- Exercise testing is essential in patients with heart failure with preserved ejection fraction to identify exercise induced pulmonary hypertension and screen for diastolic ventricular interaction.

**What Are the Clinical Implications?**

- Diastolic ventricular interaction represents a novel therapeutic target for heart failure with preserved ejection fraction patients, for whom effective therapies are currently lacking.

## Introduction

Heart failure with preserved ejection fraction (HFpEF) accounts for approximately half of all patients with heart failure.^1^ The morbidity and mortality of this syndrome is similar to that of heart failure with reduced ejection fraction (HFrEF)^2^; however, the therapeutic paradigm of blockade of maladaptive neurohumoral activation that is the cornerstone of treatment in HFrEF has not been successful in HFpEF.^3^ This likely reflects a fundamental difference in pathophysiology, explaining the lack of effective therapy for HFpEF.

While there are typically subtle abnormalities of contractile function in HFpEF despite a normal left ventricular ejection fraction,^4^ diastolic dysfunction plays the dominant role in limiting stroke volume augmentation on exercise.^5^ Current criteria for the diagnosis of HFpEF rely on the demonstration of at least moderate resting diastolic dysfunction^1^; however, we and others, have emphasized the dynamic nature of left ventricular (LV) diastolic performance during exercise.^6^, ^7^ Left ventricular active relaxation is a highly energy dependent process.^8^ In health, the rate of LV active relaxation increases during exercise, maximizing the efficacy of diastolic filling in the context of a shorter diastolic duration at high heart rates.^9^ We have previously shown that patients with HFpEF demonstrate a profound impairment, or indeed a reversal, of this physiological increase in LV active relaxation rate on exercise, because of a combination of cardiac energetic impairment and vasculo‐ventricular mismatch.^6^ This impairment of diastolic filling leads to a marked increase in left ventricular end‐diastolic pressure (LVEDP) and pulmonary artery pressure (PAP) on exercise in patients with HFpEF, even though resting LVEDP may be normal or near normal.^7^ A corollary of this is that resting diastolic dysfunction alone may not be a reliable indicator of impaired diastolic performance on exercise.^6^, ^10^

In experimental models of acute pulmonary hypertension (PHT), the associated increase in right ventricular diastolic volume stretches the pericardium, increasing pericardial pressure. Diastolic filling of the left ventricle is then constrained by the pericardium (pericardial constraint), and by the right ventricle via the interventricular septum (diastolic ventricular interaction, DVI). In an experimental model, the position and shape of the interventricular septum at end‐diastole was shown to be highly correlated with the pressure difference between the 2 ventricles at end‐diastole, which reflects the true LV “preload”.^11^ As there is normally a significant LV‐right ventricular (RV) pressure difference at end‐diastole in health, the septum is convex towards the right, but as this trans‐septal gradient falls and DVI develops, the septum becomes flatter at end‐diastole.^12^, ^13^, ^14^ The tight relationship between end‐diastolic radius of curvature and end‐diastolic trans‐septal pressure gradient has also been confirmed in the human heart.^15^ We previously demonstrated substantial DVI in an experimental model of systolic heart failure,^16^ and also important DVI in ≈40% of patients with HFrEF.^17^

We hypothesized that, similar to these acute experimental models of RV pressure overload, the acute increase in PAP occurring during exercise in patients with HFpEF would also lead to the development of DVI, further aggravating the abnormal diastolic filling in these patients and exacerbating the impairment of the Frank‐Starling mechanism.

## Methods

The data that support the findings of this study are available from the corresponding author upon reasonable request. Thirty consecutive patients with clinically stable HFpEF were recruited from a community‐based echocardiography study to investigate the incidence of HFpEF in people in the UK aged >60 years.^18^ Patients had clinical features of heart failure (New York Heart Association class II symptoms or greater), LV ejection fraction ≥50%, and echocardiographic evidence of diastolic dysfunction (and no significant valvular disease) as per the European Society of Cardiology (ESC) guidelines at the time of study initiation.^19^ All patients were invited for cardiopulmonary exercise testing. In patients with borderline diastolic parameters (resting E/E′ 8–15), the diagnosis of HFpEF required the additional confirmation of objective evidence of exercise limitation (peak oxygen consumption, VO_2_, <85% of predicted normal), and a pattern of gas exchange indicating a cardiac basis for the exercise limitation (VE/VCO_2_ slope >34, VO_2_ at anaerobic threshold <40%, and normal breathing reserve).^20^ Predicted values for peak VO_2_ for age and sex were taken from a statement on exercise standards from the writing group of the American Heart Association.^21^ No patients had left or right bundle branch block on ECG. Patients with atrial fibrillation were excluded on the basis of varying diastolic filling times, which would have introduced inaccuracies into the measurement of septal normalized radius of curvature and required averaging over numerous beats.

Age and sex matched HT controls were recruited from hypertension clinics at the Aberdeen Royal Infirmary, Aberdeen, UK, and the Norfolk and Norwich University Hospitals, Norwich, UK. Healthy volunteers were recruited from the community study and matched for age and sex. All controls underwent cardiopulmonary exercise testing and had normal exercise capacity (peak VO_2_ >90% predicted for age and sex) and normal resting echocardiograms and ECGs. Patients fasted for at least 4 hours before the exercise tests and avoided caffeine for 24 hours before the study visits. Medications were taken as usual. The study was approved by the North of Scotland National Health Service (NHS) Research Ethics Committee, Aberdeen, UK. All procedures were performed in accordance with the Declaration of Helsinki and followed written informed consent.

### Exercise Stress Echocardiography

Semi‐supine exercise stress echocardiography (Vivid E‐9, GE Healthcare, Horten, Norway) was undertaken while patients exercised using an electronically‐braked bicycle (E‐Bike ergometer EL, GE Healthcare, Horten, Norway). Peak heart rate and workload achieved during cardiopulmonary exercise testing were used to create the exercise protocol for each subject, aiming to achieve maximum heart rate in 8 to 10 minutes. Echocardiography settings were set to a low mechanical index of <0.2. Intravenous transpulmonary echocardiographic contrast (Sonovue, Bracco Imaging SpA, Colleretto Giacosa, Italy) was then given, and a resting mid‐ventricular parasternal short axis view was obtained below the mitral leaflets, just above the papillary muscle insertion points. At peak exercise, a further 1 mL Sonovue was injected, and the parasternal short axis window was recorded as above. Apical 4‐ and 2‐chamber views were also obtained at rest and peak exercise to measure LV volumes. No study participants developed significant mitral regurgitation during exercise, as assessed following lysis of microbubbles with a high mechanical index impulse.

### End‐Diastolic NRC Measurement

**Figure 1:**
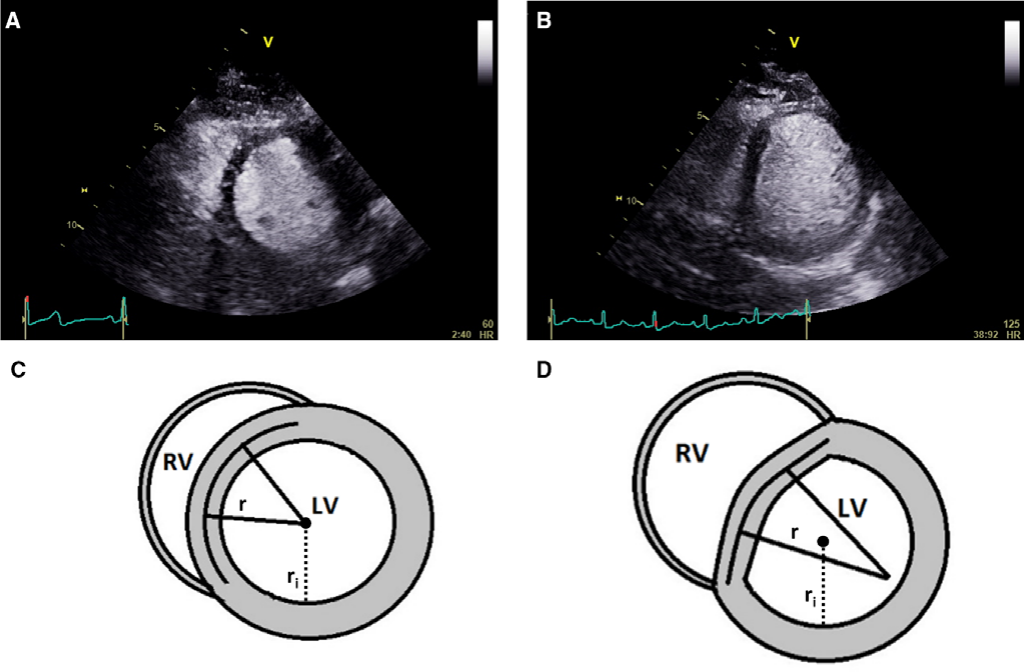
Septal flattening from diastolic ventricular interaction causes a D‐shaped left ventricle, and can be quantified from the normalized radius of curvature. **A**, A resting contrast echocardiogram in the parasternal short axis view of the left ventricle of a patient with heart failure with preserved ejection fraction, demonstrating a circular interventricular septum at end‐diastole. **B**, In the same patient during exercise, the subsequent development of pulmonary hypertension leads to pericardial constraint and diastolic ventricular interaction, flattening the septum and resulting in a D‐shaped LV. **C**, The shape of the left ventricular short axis image is calculated from the ratio of the radius extrapolated from the demarcated septum (*r*) to the cavity radius (*r*_*i*_), called the normalized radius of curvature. This is close to 1 at rest. **D**, During exercise in patients with diastolic ventricular interaction, the flattened septum produces a calculated NRC value much >1. LV indicates left ventricle; NRC, normalized radius of curvature; RV, right ventricle.

Still images of the mid‐ventricle were analyzed by 2 investigators (B.L.L. and V.S.V.) masked to the clinical status of the subject, and the mean of the measurements from these 2 investigators was used. The intra‐observer and inter‐observer variability of diastolic NRC was <10%. The normalized radius of curvature (NRC) of the left ventricle at end‐diastole was measured using the operator‐independent method described by Dong et al^22^ (Figure 1), briefly:

1. Images were exported into ImageJ/Fiji (National Institutes of Health, Bethesda, Maryland, USA);
2. Using the “Septal Radius Calculator” plug‐in, LV area (A) was measured at end‐diastole by tracing the endocardial border (with the papillary muscles included);
3. The “idealized” radius (*r*_*i*_) was then automatically calculated from A, using the equation for the area of a circle, $r_{i}=\sqrt{A/\pi }$;
4. The endocardial borders of the septum were then traced for the left ventricle, and right ventricle between the RV insertion points, to calculate the septal radius of curvature (*r*); and
5. NRC was calculated as the ratio of septal and idealized radii, *r*/*r*_*i*_.

### Measurement of Left Ventricular End‐Diastolic Volume

LV end‐diastolic volume (LVEDV) was measured in apical 4‐ and 2‐chamber windows at rest and at peak‐exercise, by the Simpson bi‐plane method.^23^

### Measurement of Normalized End‐Systolic Radius of Curvature on Exercise

While right ventricular systolic pressure (RVSP) can be measured relatively accurately at rest using the tricuspid regurgitant jet velocity plus an assumed right atrial pressure (based on collapse of the inferior vena cava during inspiration),^24^ the assumptions underlying this estimation are no longer valid on exercise, where intrathoracic pressure excursions are greatly increased.^25^ In children with pulmonary hypertension, King et al demonstrated that the LV end‐systolic trans‐septal pressure gradient tightly correlated with the inverse of the LV end‐systolic NRC (*r*/*r*_*i*_) with Pearson's *r*=0.86.^26^ End‐systolic septal radius of curvature was also previously shown in another study to be strongly related to RVSP in patients with atrial septal defect.^27^ While it is possible to estimate absolute RVSP from the formula described by King et al, because the formula has not been validated in this patient population, we instead report the systolic normalized radius of curvature measurements, which correlate with RVSP. The NRC at end‐systole was measured by 2 investigators (S.K.P. and B.L.L.) blinded to patient clinical status and the mean of the measurements was used in subsequent calculations. The intra‐observer and inter‐observer variability of systolic NRC was <10%.

### Statistical Analysis

Values are expressed as mean±SE of the mean (SEM). Baseline characteristics and outcome measures of the 3 groups were compared using ANOVA for continuous measures, with Bartlett's test to assess equality of variance. When the test was significant, each 2‐way comparison was tested using a *t* test, assuming equal variance if possible, otherwise unequal variances were used. To control the type‐I error rate, we used Bonferroni adjustment for the 2‐way comparisons, declaring significance if the *P* value was ≤0.017 (ie, 0.05÷3). The Kruskal–Wallis test was used for non‐normally distributed data, and 2‐way comparisons were made using the Mann–Whitney *U* test. Categorical variables were compared between groups using Fisher exact test. The relationship between variables was assessed using scatter plots and Pearson *r*, or Spearman rho where the data were not normally distributed. All analyses were undertaken using Stata version 14.1/SE (StatCorp, College Station, TX) and R version 3.2.3 (R Foundation for Statistical Computing, Vienna, Austria).

## Results

### Demographics

The baseline characteristics of study participants are shown in Table 1. HFpEF patients were similar to controls in terms of age, sex, and cardiovascular risk factors, but were more overweight, with an average body mass index of 29.3±0.7 kg/m^2^ (*P*<0.001). HFpEF patients also had an altered chronotropic response to exercise, and a higher percentage were on beta‐blocker and aspirin therapy. No patients were on loop diuretic therapy. There was a trend towards higher resting brain natriuretic peptide (BNP) levels in HFpEF patients, but this was not statistically significant (*P*=0.08). Left atrial volume index (LAVI) was not significantly different between HFpEF and hypertensive patients (*P*=0.27), but both were larger than Healthy controls (*P*<0.001). However, LV mass indexed to body surface area (LVMI) was greater in HFpEF patients compared with hypertensive (*P*=0.02) and Healthy controls (*P*<0.001).

**Table 1:** Baseline Characteristics

	HFpEF (n=30)	HT (n=17)	Healthy (n=17)	*P* Value
Age, y	71.9 (1.2)	69.4 (1.3)	69.2 (1.1)	0.20
Female (%)	22 (73)	7 (41)	8 (47)	0.06
White (%)	30 (100)	17 (100)	17 (100)	1.00
BMI, kg/m^2^	29.3 (0.7)	26.8 (0.8)	25.0 (0.5)	<0.001^a^
Comorbidities				
Hypertension (%)	22 (73)	17 (100)	0 (0)	<0.001^a^
Diabetes mellitus (%)	6 (20)	1 (6)	0 (0)	0.08
IHD (%)	4 (13)	0 (0)	0 (0)	0.09
NYHA Class				
1 (%)	0 (0)	17 (100)	17 (100)	<0.001^a^
2 (%)	8 (27)	0 (0)	0 (0)	
3 (%)	22 (73)	0 (0)	0 (0)	
4 (%)	0 (0)	0 (0)	0 (0)	
Medications				
Aspirin (%)	13 (43)	2 (13)	0 (0)	0.002^b^
Beta‐blocker (%)	8 (27)	1 (6)	0 (0)	0.02^b^
ACE‐I or ARB (%)	15 (50)	10 (63)	0 (0)	<0.001^a^
MRA or thiazide diuretic (%)	13 (43)	6 (38)	0 (0)	0.002^b^
Echocardiography				
LV ejection fraction, %	64.5 (1.4)	64.2 (1.7)	60.8 (1.6)	0.21
E, m/s	0.7 (0.05)	0.8 (0.07)	0.5 (0.05)	0.004^b^
A, m/s	0.9 (0.05)	0.9 (0.05)	0.7 (0.02)	<0.001^a^
E/A	0.8 (0.03)	0.9 (0.02)	0.8 (0.05)	0.36
Mitral deceleration time, ms	262.4 (17.5)	242.0 (11.4)	210.5 (10.0)	0.052
E′_lateral_, cm/s	7 (6, 9)	8 (8, 9)	8 (8, 9)	0.53
E/e′_average_	11 (9, 16)	9 (8, 10)	8 (6, 9)	<0.001^a^
LA volume index, mL/m^2^	42.2 (3.8)	52.5 (4.2)	28.8 (2.5)	<0.001^a^
LVMI, g/m^2^	96.5 (4.5)	72.2 (4.9)	64.1 (3.7)	<0.001^a^
BNP, pg/mL	60.9 (22.1, 93.1)	13.6 (11.3, 39.4)	15.7 (12.3, 54)	0.08
Exercise				
Resting heart rate, bpm	75.5 (2.0)	78.6 (2.7)	73.2 (2.6)	0.35
Peak exercise heart rate, bpm	112.1 (3.2)	121.9 (3.2)	128.7 (3.1)	0.003^b^
Resting diastolic BP, mm Hg	83.7 (2.4)	83.2 (2.4)	81.7 (2.6)	0.85
Resting systolic BP, mm Hg	147.2 (3.7)	144.2 (4.2)	128.4 (4.6)	0.007^b^
Peak diastolic BP, mm Hg	84.4 (2.8)	87.9 (2.4)	88.2 (3.6)	0.62
Peak systolic BP, mm Hg	170.9 (5.2)	195.3 (5.3)	201.3 (8.0)	0.001^b^
Exercise duration, min	10.9 (0.6)	13.5 (0.8)	11.4 (0.3)	0.12
FEV1, L	2.24 (0.10)	2.40 (0.24)	2.46 (0.12)	0.17
Peak VO_2_/predicted peak VO_2_, %	61.1 (2.3)	97.6 (3.0)	98.4 (1.7)	<0.001^a^
VO_2_ at AT/predicted peak VO_2_, %	34.2 (0.9)	64.3 (2.0)	56.5 (2.4)	<0.001^a^
VE‐VCO_2_ at AT	38.7 (0.9)	29.9 (1.1)	27.6 (0.6)	<0.001^a^
Respiratory exchange ratio	1.13 (0.02)	1.14 (0.02)	1.11 (0.01)	0.49

Values are mean (SEM) or median (interquartile range). A indicates atrial flailing velocity; ACE‐I, angiotensin converting enzyme inhibitor; AF, atrial fibrillation; ARB, angiotensin receptor blocker; AT, anaerobic threshold; BMI, body mass index; BNP, brain natriuretic peptide; BP, blood pressure; E, early transmitral flow velocity; E′, mitral annular velocity; FEV1, forced expiratory volume over 1 second; HFPEF, heart failure with preserved ejection fraction; HT, hypertensive control; IHD, ischemic heart disease; LV, left ventricle; MRA, mineralocorticoid receptor antagonist; NYHA, New York Heart Association; VCO_2_, carbon dioxide production; VE, minute ventilation; VO_2_, oxygen consumption.

a *P*<0.001.

b *P*<0.05.

### End‐Diastolic NRC

**Figure 2:**
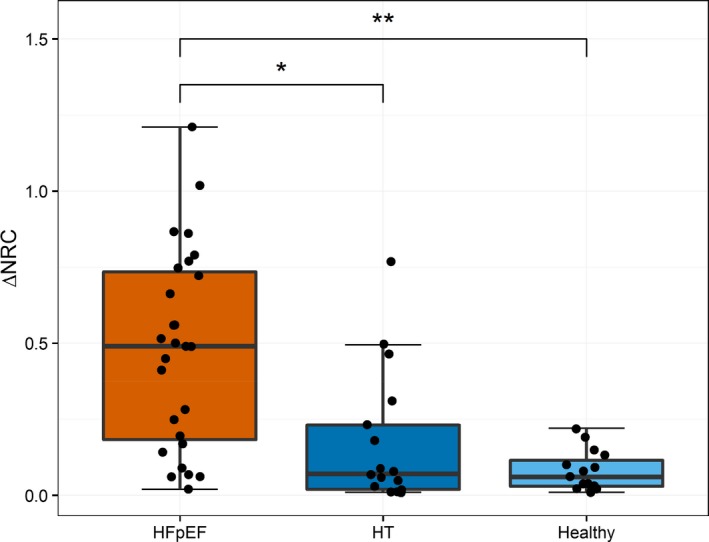
Change in end‐diastolic normalized radius of curvature with exercise by patient group. Boxplots show the change in NRC with exercise, with raw data overlaid. The change in end‐diastolic NRC with exercise was significantly higher in HFpEF patients compared with normotensive (HFpEF vs healthy, *P*<0.001) and hypertensive controls (HFpEF vs HT,* P*=0.002). There was a trend towards a difference between HT and healthy controls, but this was not statistically significant (*P*=0.07). ^†^ *P*<0.001, *P<0.05. HFpEF indicates heart failure with preserved ejection fraction; HT, hypertensive controls; NRC, normalized radius of curvature.

As shown in Table 2, resting end‐diastolic NRC was similar across all 3 groups (*P*=0.80). At peak exercise, NRC increased significantly in HFpEF patients only (ΔNRC, +0.45±0.06, *P*<0.001), with non‐significant increases observed in hypertensive (+0.17±0.05, *P*=0.38) and Healthy controls (+0.06±0.03, *P*=0.93). ΔNRC was greater in HFpEF compared to both hypertensive (*P*=0.002) and Healthy controls (*P*<0.001). Peak NRC was higher in the HFpEF patients (1.54±0.07) compared with hypertensive (1.27±0.06, *P*=0.004) and healthy controls (1.14±0.03, *P*<0.001). Although there was a trend towards a higher NRC on exercise in hypertensive versus healthy controls (*P*=0.05) this was not statistically significant following the pre‐specified Bonferroni's adjustment (Figure 2, Table 2). In HFpEF patients, peak NRC also correlated with LVMI (*r*=0.33, *P*<0.05).

**Table 2:** End‐Diastolic NRC at Rest and Peak Exercise

	HFpEF (n=30	HT (n=17)	Healthy (n=17)	*P* Value (Overall)	*P* Values for 2‐Way Comparisons if Overall Significant		
					HFpEF vs HT	HFpEF vs Healthy	HT vs Healthy
NRC (dias) rest	1.09 (0.01)	1.10 (0.02)	1.09 (0.02)	0.80			
NRC (dias) peak exercise	1.54 (0.07)	1.27 (0.06)	1.14 (0.03)	0.0001	0.006^a^	0.0001^a^	0.241
ΔNRC (dias)^b^	0.45 (0.06)	0.17 (0.05)	0.06 (0.03)	0.0001	0.0014^a^	0.0001^a^	0.472

Values are mean (SEM). HFpEF indicates heart failure with preserved ejection fraction; HT, hypertensive controls; NRC, normalized radius of curvature; ΔNRC, change in normalized radius of curvature.

a *P*_(adjusted)_≤0.017.

b The change in end‐diastolic NRC on exercise was significant for HFpEF patients (*P*<0.001), but not hypertensive (*P*=0.38) or normotensive controls (*P*=0.93).

### End‐Systolic NRC

Resting end‐systolic NRC was higher in HFpEF patients than hypertensive controls (1.21±0.02 versus 1.16±0.02), and higher in hypertensive controls compared with healthy controls (1.16±0.02 versus 1.11±0.02), implying higher resting right ventricular systolic pressures. However, this difference was statistically significant between HFpEF and Healthy only (*P*=0.002; Table 3). At peak exercise, end‐systolic NRC was higher in HFpEF patients (1.47±0.05), and this was significantly higher than hypertensive and Healthy controls (*P*=0.0049 and *P*=0.011, respectively).

**Table 3:** End‐Systolic NRC at Rest and Peak Exercise

	HFpEF (n=30)	HT (n=17)	Healthy (n=17)	*P* Value (Overall)	*P* Values for 2‐Way Comparisons if Overall Significant		
					HFpEF vs HT	HFpEF vs Healthy	HT vs Healthy
NRC (sys) rest	1.21 (0.02)	1.16 (0.02)	1.11 (0.02)	0.004	0.092	0.002^a^	0.032
NRC (sys) peak exercise	1.47 (0.05)	1.31 (0.04)	1.19 (0.02)	0.0001	0.0049^a^	0.0001^a^	0.011^a^
ΔNRC (sys)^b^	0.26 (0.03)	0.15 (0.04)	0.08 (0.03)	0.0015	0.022	0.0005^a^	0.428

Values are mean (SEM). HFpEF indicates heart failure with preserved ejection fraction; HT, hypertensive controls; NRC, normalized radius of curvature.

a *P*_(adjusted)_≤0.017.

b End‐systolic NRC increased in all groups with exercise; this was significantly greater in HFpEF patients compared with hypertensive and healthy controls.

### End‐Diastolic NRC and End‐Systolic NRC

**Figure 3:**
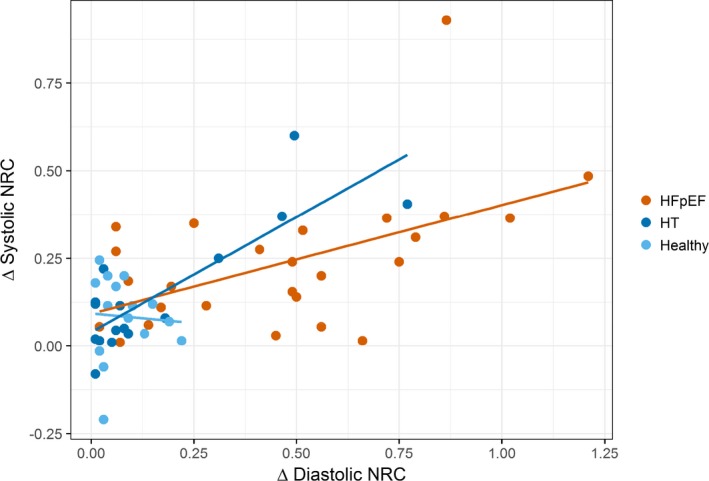
Change in systolic normalized radius of curvature (NRC) vs diastolic NRC with exercise. A scatter plot with individual linear best‐fit lines for each group. There was a positive correlation between the change in end‐diastolic NRC and change in systolic NRC in HFpEF patients (ρ=0.51, *P*=0.007), representing a greater increase in right ventricular systolic pressure in those patients with increasing diastolic NRC with exercise. A positive correlation was also seen in hypertensive controls (ρ=0.78, *P*=0.01), but not in healthy controls (ρ=−0.14, *P*=0.61). HFpEF indicates heart failure with preserved ejection fraction; HT, hypertensive controls; NRC, normalized radius of curvature.

There was a positive correlation between peak diastolic NRC and systolic NRC on exercise in HFpEF (ρ=0.51, *P*=0.007) (Figure 3). A positive correlation was also seen in hypertensive controls (ρ=0.60, *P*=0.01), but there was no significant correlation in healthy controls (ρ=−0.14, *P*=0.61).

### End‐Diastolic NRC and Cardiac Volumes

**Figure 4:**
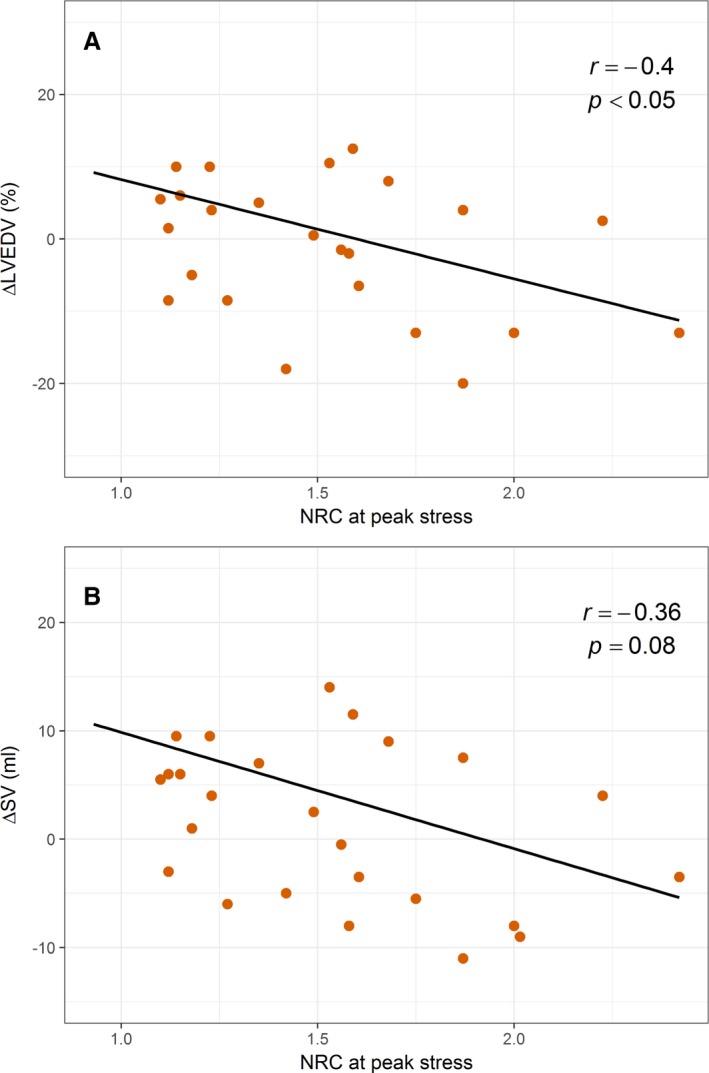
Change in LV volumes vs peak diastolic normalized radius of curvature on exercise in HFpEF patients. Scatter plots with linear best‐fit lines. **A**, There was a modest negative correlation between peak end‐diastolic NRC and the change in LVEDV (∆LVEDV) on exercise in HFpEF patients (*r*=−0.40, *P*=0.046). **B**, There was also a trend towards a negative correlation between peak NRC and the change in SV (∆SV) with exercise in HFpEF patients, however this did not reach statistical significance (*r*=−0.36, *P*=0.08). HFpEF indicates heart failure with preserved ejection fraction; HT, hypertensive controls; LVEDV, left ventricular end diastolic volume; NRC, normalized radius of curvature; SV, stroke volume.

LVEDV was similar at rest in all 3 groups (Table 4). LVEDV increased with exercise in Healthy controls (ΔLVEDV, +8.8±2.0 mL, *P*<0.001) and in hypertensive controls (+6.1±2.4 mL, *P*=0.001). However, LVEDV failed to increase appropriately during exercise in HFpEF (+1.5±1.8 mL, *P*=0.71), and indeed fell in 13 patients. The change in LVEDV was significantly different between HFpEF and healthy (*P*<0.001) and HFpEF and hypertensive (*P*=0.014) controls. In the HFpEF patient group, there was a modest negative correlation between ΔLVEDV and peak NRC (*r*=−0.40, *P*=0.046; Figure 4A).

**Table 4:** Cardiac Volume Analysis at Rest and Peak Exercise

	HFpEF (n=30)	HT (n=17)	Healthy (n=17)	*P* Value (Overall)	*P* Values for 2‐Way Comparisons if Overall Significant		
					HFpEF vs HT	HFpEF vs Healthy	HT vs Healthy
LVEDV rest, mL	91.6 (3.1)	104.1 (5.0)	90.5 (3.9)	0.04	0.03	0.83	0.04
LVEDV peak exercise, mL	93.1 (3.3)	111.5 (5.2)	99.3 (4.0)	0.008	0.003^a^	0.24	0.07
ΔLVEDV, mL	1.5 (1.8)	6.1 (2.4)	8.8 (2.0)	0.001	0.01^a^	0.0006^a^	0.38
SV rest, mL	59.5 (2.0)	67.6 (3.1)	59.3 (2.5)	0.048	0.03	0.95	0.05
SV peak exercise, mL	63.5 (2.2)	78.2 (3.7)	70.0 (2.6)	0.001	0.0007^a^	0.06	0.08
ΔSV, mL	1.0 (1.4)	9.6 (1.8)	10.7 (1.7)	<0.0001	<0.001^a^	<0.001^a^	0.66

Values are mean (SEM). HFpEF indicates heart failure with preserved ejection fraction; HT, hypertensive controls; LVEDV, left ventricular end‐diastolic volume; SV, stroke volume; ΔLVEDV (mL), LVEDV peak exercise−LVEDV rest; ΔLVEDV, change in left ventricular end‐diastolic volume; ΔSV (mL), SV peak exercise−SV rest. ΔSV, change in stroke volume.

a *P*_(adjusted)_≤0.017. Patients with HFpEF failed to increase end diastolic volume and therefore stroke volume with exercise, in contrast to hypertensive and healthy controls.

Stroke volume (SV) was also similar at rest in all 3 groups (Table 4). SV increased in healthy (ΔSV, +10.7±1.7 mL, *P*<0.0001) and hypertensive controls (+9.6±1.8 mL, *P*<0.0001) on exercise, but failed to increase appropriately in HFpEF (+1.0±1.4 mL, *P*=0.05). Indeed, SV fell in the 13 HFpEF patients who failed to increase their LVEDV. In HFpEF patients, ΔSV trended towards a modest negative correlation with ΔNRC (*r*=−0.36, *P*=0.08; Figure 4B). There was also a trend towards a positive correlation between peak heart rate and ΔSV in the whole patient group (n=64; *r*=0.22, *P*=0.09), however this was lost when considering the HFpEF group alone (*r*=−0.05, *P*=0.79).

## Discussion

In this study, we show that, during exercise, marked flattening of the interventricular septum occurs at end‐diastole in HFpEF patients, but not in hypertensive or normotensive controls, indicating the development of DVI. The degree of DVI was modestly inversely correlated with the increase in LVEDV on exercise. Our findings suggest that DVI is an important mechanism aggravating the pre‐existing diastolic abnormality in HFpEF, contributing to the limitation of utilization of the Frank‐Starling mechanism on exercise, and to exercise limitation.

HFpEF is poorly understood, and no established therapies exist, despite its estimated economic burden of $54 billion annually worldwide.^28^ The HFpEF syndrome represents a range of underlying etiologies and these may present clinically as a number of sub‐phenotypes.^29^ These differing sub‐phenotypes are likely responsible for the heterogeneity in response to interventions^30^, ^31^ and, by extension, the disappointing results of clinical trials in the area to date. This has prompted many to advocate for sub‐phenotype‐based treatment regimens to improve patient outcomes^32^ and for careful and thorough characterization of patients enrolled in studies involving patients with HFpEF. Primary abnormalities of LV active relaxation, increased passive LV stiffness (because of myocardial fibrosis, glycation of collagen, titin isoform shift and impaired titin phosphorylation), and impaired atrial mechanical function, may each contribute to impaired diastolic filling at rest in HFpEF.^5^, ^33^ This results in raised LVEDP and an impaired ability to use the Frank‐Starling mechanism to increase stroke volume. However, as we have previously demonstrated, there is profound dynamic impairment of LV active relaxation during exercise,^4^, ^6^ even in patients with mild elevations of LVEDP at rest. The Mayo group have therefore advocated exercise hemodynamic studies in the diagnosis and evaluation of patients with the endophenotype of near‐normal resting LVEDP.^7^ Others have advocated stress echocardiography to assess changes in E/E′ to estimate dynamic changes in LVEDP.^34^

PHT is common in HFpEF, being present in >80% of patients and strongly associated with mortality in a community‐based study of 244 patients.^35^ While much of this PHT is accounted for by post‐capillary factors, a significant number of patients also demonstrate a pre‐capillary component, because of remodeling of the pulmonary arterioles.^36^ PAP increases substantially on exercise in association with substantial increases in LVEDP, even in some patients without significantly raised LVEDP or PAP at rest.^7^ This results in abnormal RV‐pulmonary artery coupling with exercise in HFpEF, and associated substantial elevation of right atrial pressure attributable to RV dysfunction.^37^, ^38^ Our data are consistent with these studies, showing that the end‐systolic NRC (correlated with RVSP) was moderately increased at rest, but much more markedly increased on exercise in HFpEF versus controls. Our findings are consistent with, but extend those of a recent paper in which HFpEF patients with a pulmonary hypertension sub‐phenotype, particularly those with a pre‐capillary component, were shown to develop a paradoxical fall in LV end diastolic transmural pressure gradient on exercise.^39^ Our study shows that even patients with only mildly increased resting end‐systolic NRC (implying mildly increased resting RVSP) may develop severe DVI on exercise.

In health, pericardial pressure is little more than zero, and the end‐diastolic pressure of the thin‐walled RV is similar.^40^ However, the pericardium exhibits a J‐shaped stress‐strain relationship, and pericardial pressure (and right ventricular end diastolic pressure) markedly increases as the pericardium becomes stretched.^41^ Thus, in experimental models of acute PHT, such as pulmonary artery banding or massive pulmonary embolism, pericardial pressure and right ventricular end diastolic pressure become markedly increased.^42^, ^43^ In this setting, even though LVEDP may be raised, LV filling is constrained by the pericardium and by the RV through the interventricular septum which becomes flattened at end‐diastole (DVI). The pericardium can grow in response to chronic cardiac enlargement, which prevents the development of pericardial constraint, at least at rest.^41^ Nevertheless, we showed that in chronic HFrEF ≈40% of patients exhibited evidence of DVI at rest, presumably because of progressive enlargement of the ventricles.^17^ Recently, Obokata et al demonstrated DVI in an obese (body mass index ≥35 kg/m^2^) HFpEF patient population.^44^ They showed that, compared with a non‐obese HFpEF group and controls, obese HFpEF patients displayed plasma volume expansion and right ventricular dilatation, and had greater RV dysfunction and biventricular remodeling. DVI was apparent at rest in the obese HFpEF patients, but not in the non‐obese HFpEF or control groups, and this increased with rising pulmonary artery systolic pressure with exercise, likely contributing to the profound exercise intolerance in this group.^44^

In the current study, we confirmed our hypothesis that the development of PHT on exercise would lead to the development of DVI in patients with HFpEF. At rest, the end‐diastolic NRC was similar in patients and hypertensive and normotensive controls, and was close to 1, indicating that the left ventricle was almost circular in the short axis; however, on exercise it substantially increased in patients with HFpEF but not in the control groups. These observations indicate a reduction in the end‐diastolic trans‐septal pressure gradient, reducing the effective LV preload and, via the Frank‐Starling mechanism, the stroke volume. Consistent with this, LVEDV increased during exercise in normotensive and hypertensive controls, but remained unchanged in patients. Indeed, LVEDV fell in 13 HFpEF patients. The change in LVEDV during exercise exhibited a modest negative correlation with peak end‐diastolic NRC on exercise, and a trend was seen with SV, indicating contribution of the development of DVI to the loss of the Frank‐Starling mechanism on exercise. While the mean change in LVEDV on exercise was close to zero in patients, the standard deviation was wide, indicating substantial heterogeneity in response (Table 4). It is noteworthy that some of the hypertensive population had “intermediate” resting E/E′ values, comparable with those seen in many of the HFpEF patients. These patients had no evidence of exercise limitation on cardiopulmonary exercise testing and did not develop end diastolic septal flattening on exercise. This emphasizes the dramatic worsening of diastolic function on exercise in this HFpEF sub‐phenotype, leading to dramatically different exercise physiology from that of this hypertensive subpopulation. Importantly, our patients do not fit into the obese HFpEF sub‐phenotype, where DVI has been demonstrated previously^44^; they did not have overt volume overload or raised filling pressures at rest, and no resting DVI. Our study complements the findings of others in patients with the HFpEF with pulmonary vascular disease phenotype^39^ by directly demonstrating the development of septal shift on exercise.

These observations may have therapeutic implications. For example, agents that can reduce PAP, particularly during exercise, may be beneficial. We have shown that short‐term intravenous sodium nitrite therapy (50 μg/kg per minute for 5 minutes) improves hemodynamics by increasing stroke volume and reduces pulmonary vascular resistance without significant effects on mean arterial pressure in patients with HFrEF.^45^ This improvement in SV correlated with increasing LV trans‐septal pressure gradient, suggesting potential relief of pericardial constraint and DVI. In a recent randomized controlled trial,^46^ nebulized nitrite failed to increase exercise capacity, despite previous evidence showing that it improved rest and exercise hemodynamics in HFpEF.^47^ However, nebulized nitrite has a short half‐life, whereas oral inorganic nitrate supplementation causes a much more sustained increase in plasma nitrite.^48^ Indeed in a small study, 1 week of beetroot juice (containing 12.9 mmol inorganic nitrate daily) improved submaximal exercise endurance in patients with HFpEF.^49^

### Study Limitations

The tricuspid regurgitant jet velocities were not obtained in a sufficient number of patients in our study to give estimations of RVSP. Independently of this, as discussed already, the use of tricuspid regurgitant jet velocities with exercise‐stress echocardiography to estimate RVSP is problematic, and rendered invalid because of marked swings in intrathoracic pressure which preclude estimation of RA pressure.^25^ Similarly, non‐invasive measures of pulmonary vascular resistance, which provide important information in the diagnosis of PHT, also require tricuspid regurgitant jet velocities^50^ and were not obtained in this study. Invasive measures during right heart catheterization are considered to be the “gold standard” for the diagnosis of PHT at rest.^25^ However, despite evidence that exercise‐derived invasive measures to estimate LV filling pressure improve the diagnosis of exercise PHT in HFpEF,^7^ their use requires standardization and further assessment in this cohort.^51^ Additionally, the use of invasive measures in elderly hypertensive and healthy controls would not have been possible because of ethical considerations. Kingma et al demonstrated in a canine model that the end systolic NRC was tightly correlated with end‐systolic transmural pressure gradient.^52^ An equation was developed by King et al using data from children with congenital heart disease and healthy controls to estimate RVSP from end‐systolic NRC.^26^ However, this formula has not been validated in an HFpEF patient population. It is conceivable that the slope of the relationship between NRC and RVSP could differ if systolic LV stiffness (end systolic elastance) differed. We have previously shown that whilst resting LV systolic elastance was increased in HFpEF patients versus controls, it failed to increase on exercise appropriately, and exercise LV end systolic elastance was therefore similar in HFpEF patients and controls.^6^ Nevertheless we have presented data for end systolic NRC rather than deriving RVSP in order to make no assumptions.

Catecholamines, such as those released with exercise, have been shown to diminish the correlation between E/E′ and LV filling pressures.^53^ RV functional assessment and RV‐PA coupling were also not available in our study, and would have likely required invasive measurements, as non‐invasive measures have not been tested on exercise and require estimations of exercise pulmonary artery systolic pressure.^54^ In addition, targeted ischemia detection with coronary angiography was not performed in our patient cohort before enrollment, however, none of the patients had current symptoms of angina or a prior history of ischemic events, none had ECG changes detected on exercise testing, and none developed wall motion abnormalities during the stress echocardiogram, making reversible ischemia unlikely. Finally, as the HFpEF patients had a slightly higher body mass index, it is not possible to know whether this may have played any role in our findings. However, we feel this would be unlikely given the small difference in body mass index. Likewise, we note the small differences in some medication use and echocardiographic parameters between the groups. Our sample size did not allow us to undertake further adjustments, however, as these patients were recruited from the community and not fluid overloaded at the time of the study, we would not anticipate that the small changes seen could have influenced the overall results.

## Conclusions

Substantial end‐systolic flattening of the septum (implying severe PHT) develops on exercise in a substantial proportion of HFpEF patients, resulting in DVI and pericardial constraint. LVEDP during exercise is increased in HFpEF because of dynamic slowing of LV active relaxation, together with increased passive LV stiffness.^6^ The development of DVI in patients aggravates the diastolic filling abnormality and prevents use of the Frank‐Starling mechanism to increase stroke volume on exercise. This has potential therapeutic implications, potentially with agents that target exercise pulmonary artery pressure.

## Sources of Funding

This work was funded by the British Heart Foundation, project grant reference PG/13/4/29811.

## Disclosures

None.
